# Partial Anomalous Pulmonary Venous Return Presenting in Adults: A Case Series With Review of Literature

**DOI:** 10.7759/cureus.8388

**Published:** 2020-06-01

**Authors:** Venkata Satish Pendela, Bryan E-Xin Tan, Medhat Chowdhury, Ming Chow

**Affiliations:** 1 Internal Medicine, Rochester General Hospital, Rochester, USA; 2 Pulmonary and Critical Care Medicine, Rochester General Hospital, Rochester, USA

**Keywords:** pulmonary circulation, pulmonary hypertension, cardiac magnetic resonance, anomalous venous return, adult congenital heart disease (achd)

## Abstract

Partial anomalous pulmonary venous return (PAPVR) is a congenital anomaly in which some of the pulmonary veins drain erroneously into the superior vena cava (SVC) or directly into the right atrium (RA). We present four cases of PAPVR presenting in adults. We discussed various presentations, diagnostic approaches and challenges in the management of these patients. Our first case had anomalous drainage from the right upper lobe of lung to SVC and was managed medically with riociguat and ambrisentan. The second patient had an unsuccessful attempt at repair of the anomalous vein. Our other two patients had right upper lobe veins draining into SVC. One of them had a successful surgical repair whereas the other patient declined surgery and is being monitored. In PAPVR patients, the decision for surgical repair depends on symptoms, shunt fraction, recurrent pulmonary infections, and concurrent indication for cardiac surgery.

## Introduction

Partial anomalous pulmonary venous return (PAPVR) is a congenital condition that occurs due to the failure of regression of primitive lung drainage. As a result, one or more, but not all, of the pulmonary veins drain directly into the right atrium or indirectly through a systemic vein. Clinical presentation may vary depending on the degree of left-to-right shunting. We present four cases highlighting the variable characteristics and respective treatment approaches.

## Case presentation

Case 1

**Figure 1:**
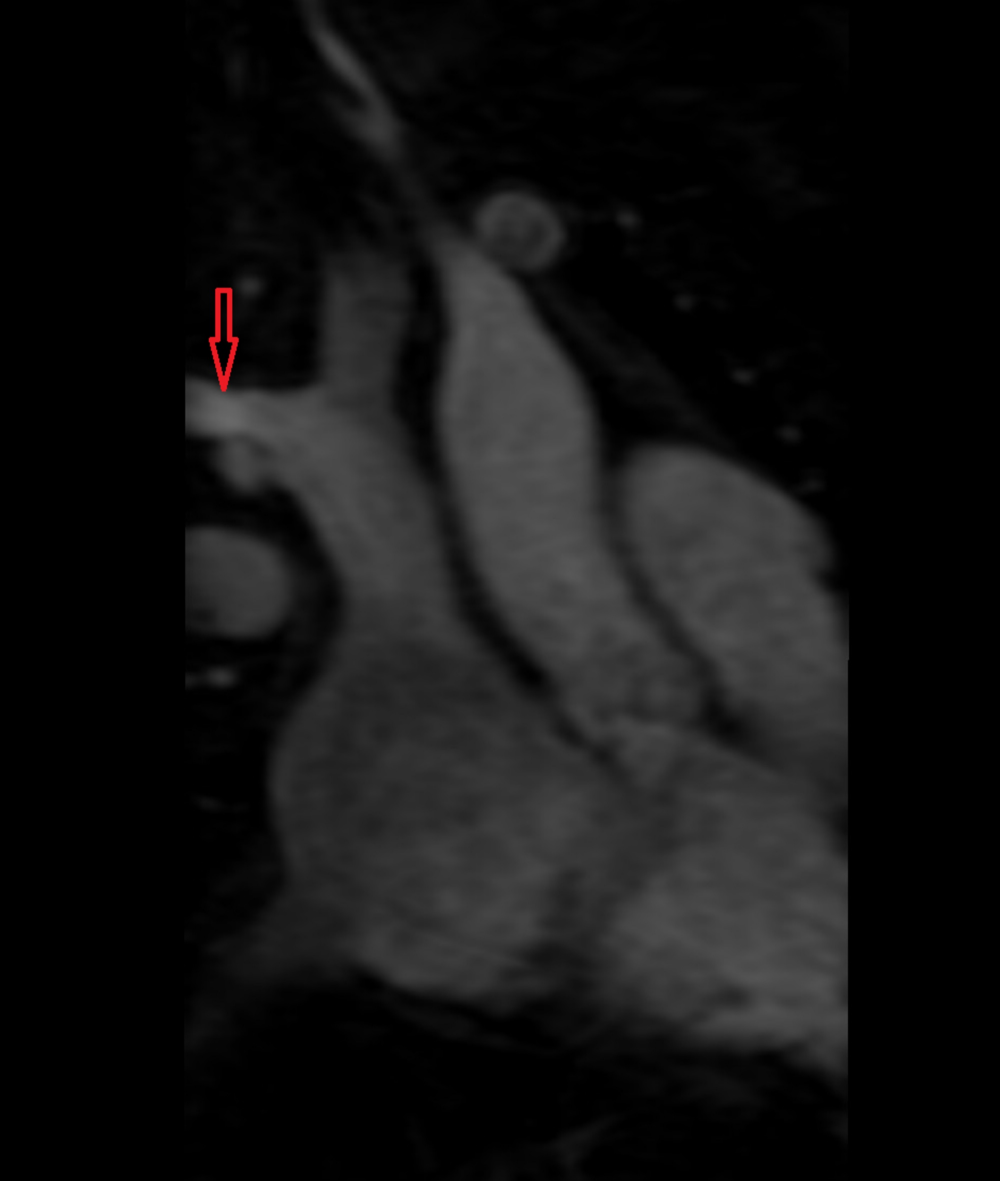
Anomalous vein arising from the right upper lobe of the lung draining into superior vena cava

A 63-year-old female with well-controlled hypertension presented to the emergency department with worsening shortness of breath and bilateral lower extremity swelling for four weeks. Her symptoms had progressed to a point where she could not walk even a block without getting short of breath. Her heart rate was 96 beats per minute and blood pressure was 150/80 mmHg. Physical examination revealed a systolic murmur in pulmonary and tricuspid areas. Transthoracic echocardiogram (TTE) showed right ventricular dilation, hypokinesia, and severe tricuspid regurgitation with pulmonary artery hypertension (right ventricular systolic pressure of 40 mmHg). Coronary angiogram showed mild non-obstructive coronary artery disease. Right heart catheterization showed severe pulmonary hypertension and evidence of severe left-to-right shunt at the atrial level with possible anomalous pulmonary vein draining into the right atrium (Table 1). A cardiac magnetic resonance imaging (MRI) subsequently confirmed the presence of PAPVR from the entire right upper lobe with drainage into the superior vena cava (SVC) and the right atrial (RA) junction with Qp/Qs ratio of 1.35 (Figure 1). A decision was made to proceed with medical management. She was initially started on tadalafil and ambrisentan. Due to persistent symptoms, tadalafil was replaced by Riociguat with significant improvement in her symptoms. A right heart catheterization performed six months later revealed a decrease in mean pulmonary artery pressures. Repeat cardiac MRI in one year showed a Qp/Qs ratio of 1.8, and she is being followed closely.

**Table 1:** Patient characteristics and findings ASD - atrial septal defect, mPAP- mean pulmonary artery pressure, PA - pulmonary artery, PCWP - pulmonary capillary wedge pressure, RA - right atrium, RHC - right heart catheterization, RV- right ventricle.

Case	Age/Sex	RHC hemodynamics	Anomalous vein arising from	Draining into	Number of anomalous pulmonary veins	Atrial septal defect/ Patent foramen ovale	Other cardiac conditions	Qp/Qs	Treatment
1	63 F	RA 19/23mmHg RV 87/23mmHg PA 88/45mmHg mPAP 59mmHg PCWP 21/18mmHg	right upper lobe of the lung	superior vena cava	two	None	None	1.35	Medical management with riociguat and ambrisentan
2	51 M	RA 14/15 mmHg RV 39/15 mmHg PA 33/20 mmHg mPAP 24mmHg PCWP 16/18 mmHg	left upper lobe of the lung	left brachiocephalic vein	two	None	Triple vessel coronary artery disease	1.2	Coronary artery bypass grafting with unsuccessful attempt for PAVPR repair, followed by conservative management.
3	42 F	RA 6mmHg RV 42/1mmHg PA 44/15mmHg mPAP 27mmHg PCWP 14mmHg	right upper lobe of the lung	superior vena cava	one	Sinus venosus ASD	None	4	Successful surgical repair of PAPVR and ASD.
4	66 M	Patient declined RHC	right upper lobe of the lung	superior vena cava	two	Sinus venosus ASD	None	2.1	Declined further workup. Conservative management.

Case 2

**Figure 2:**
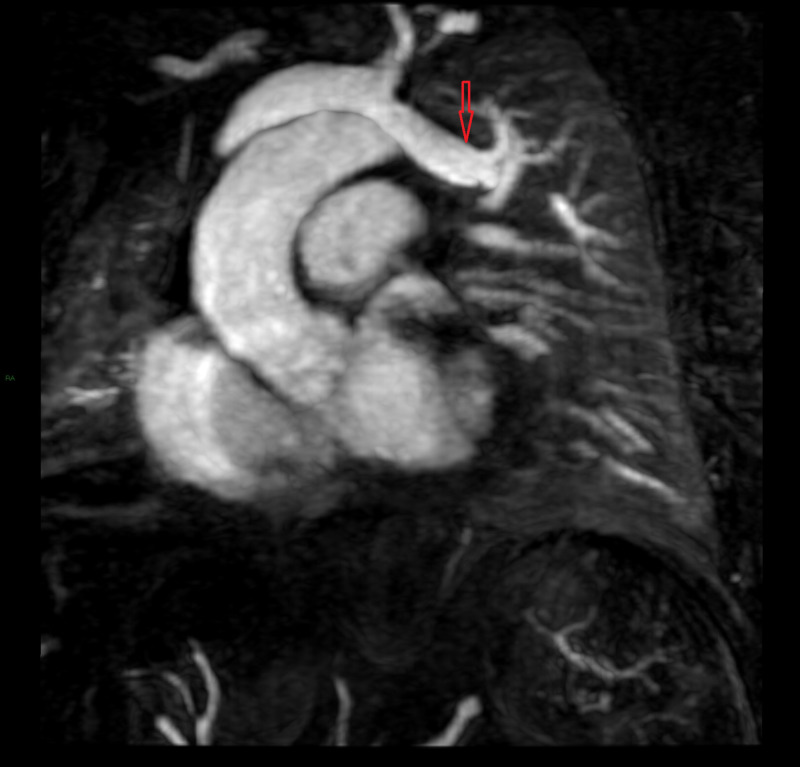
Anomalous vein arising from left upper lobe of the lung draining into left brachiocephalic vein

A 51-year-old male with a history of coronary artery disease and tobacco abuse (twenty pack-year) presented to the emergency department with worsening dyspnea on exertion over six months and worsening chest pain for one week. His chest pain was non-exertional, described as sharp, and intermittent. Blood pressure was 130/70 mmHg. Physical examination was notable for bilateral ankle edema. Spirometry was normal. TTE showed right ventricular enlargement (right ventricular end-diastolic diameter of 4.5 cm). Coronary angiogram revealed severe triple vessel disease. Right heart catheterization was consistent with anomalous left upper pulmonary venous return, as seen in Table 1. Cardiac MRI showed that the left upper lobe of the lung was draining into the left brachiocephalic vein (Figure 2). Qp/Qs ratio was 1.2. He underwent coronary artery bypass grafting (CABG), during which the anomalous vein repair was attempted, but was aborted due to the risk of injury to the phrenic nerve. We decided to pursue conservative management with close follow up.

Case 3

**Figure 3:**
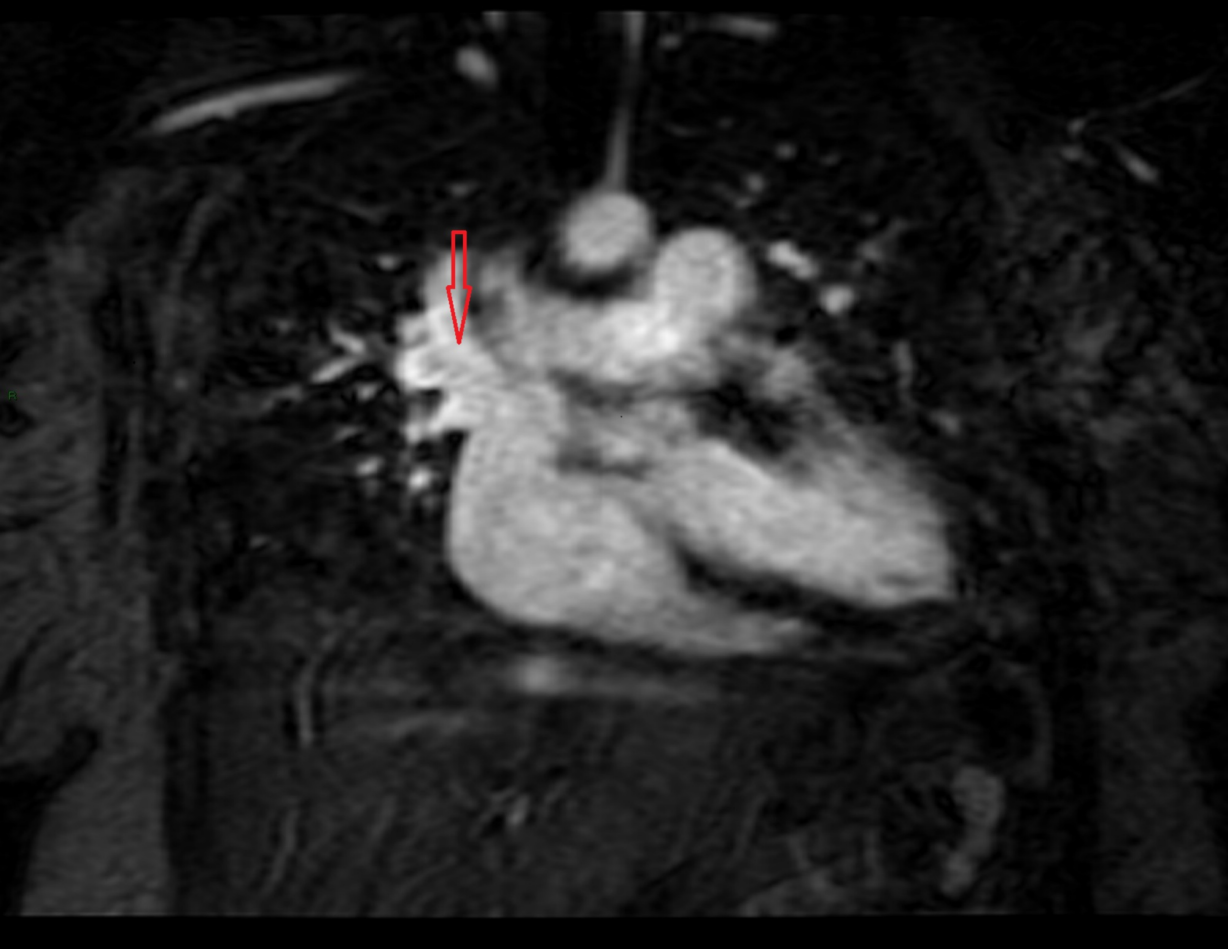
Anomalous vein arising from right upper lobe of the lung draining into superior vena cava

A 42-year-old morbidly obese female presented to the office for a pre-operative evaluation for bariatric surgery. She complained of occasional palpitations and mild sub-sternal discomfort on exertion, which relieved with rest. On physical examination, severe central obesity with pedal edema and abdominal distention was noted. A Grade 2/6 systolic murmur was heard at the left lower and upper sternal border. EKG was significant for a new-onset incomplete right bundle branch block. TTE revealed biatrial enlargement (right greater than left) and moderate pulmonic regurgitation with grade 2 diastolic dysfunction of the left ventricle. CT of the chest was notable for enlarged pulmonary vasculature. Cardiac MRI was significant for superior sinus venosus atrial septal defect (ASD) with a partial anomalous pulmonary venous connection between the right upper lobe of the lung and the superior vena cava (Figure 3). There was a significant shunting with a Qp/Qs ratio of 4. Subsequently, she underwent successful surgical repair of sinus venosus ASD and PAPVR. At follow up visits she remained asymptomatic and underwent bariatric surgery with good results.

Case 4

**Figure 4:**
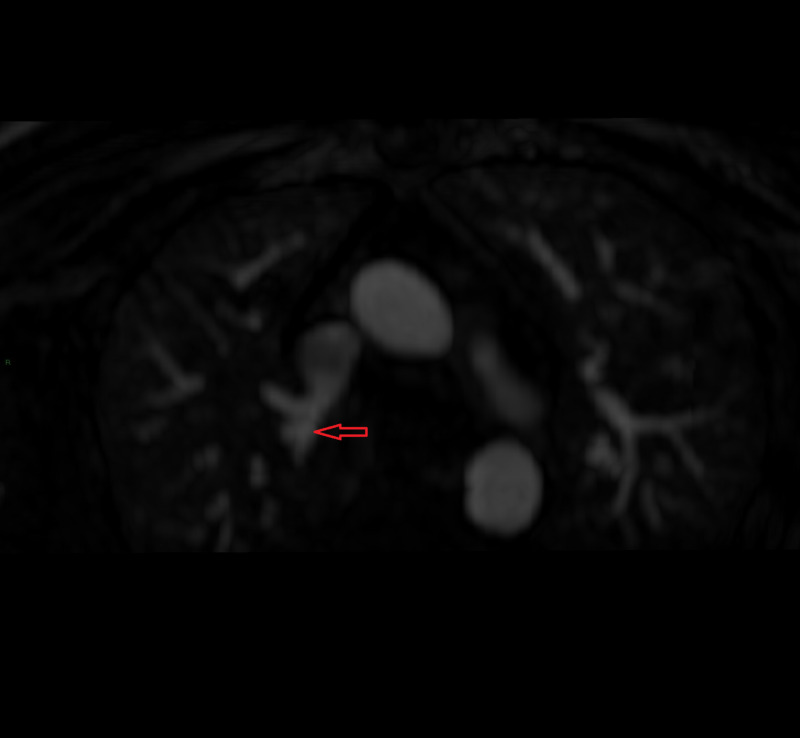
Anomalous vein arising from right upper lobe of the lung draining into superior vena cava

A 66-year-old male with a prior history of transcatheter atrial septal defect repair at age five presented to the emergency department with progressively worsening intermittent stabbing chest pain of three-day duration. He was found to be tachycardic. The electrocardiogram showed supraventricular tachycardia with a heart rate of 220 bpm. Troponin was elevated at 0.75 ng/mL (normal: 0.00 - 0.09). He was cardioverted successfully back to sinus rhythm. His coronary angiogram was unremarkable. The electrophysiological study did not disclose any inducible arrhythmia. A cardiac MRI revealed two anomalous pulmonary veins. The first anomalous vein originated from the right upper lobe and drained into the superior vena cava at approximately 5 cm above the SVC-RA junction and measured about 1 cm X 0.8 cm at the ostium. The second anomalous pulmonary vein on the right joined the SVC at the SVC-RA junction and measured 0.6 cm X 1.5 cm (Figure 4). A superior sinus venosus ASD was also suspected and there was evidence of significant shunting with Qp/Qs ratio of 2.1. The patient was offered further testing to delineate the extent of the shunting, however, he was not interested. 

## Discussion

PAPVR is a congenital anomaly in which one or more, but not all, of the pulmonary veins, return directly to the right atrium or indirectly through a systemic vein. The overall incidence of PAPVR is 0.7% based on an autopsy series [1]. However, a more recent retrospective study examining chest CT showed a disease prevalence of 0.1% [2].

PAPVR can involve one or both sides [3]. The anomalous vein most commonly connects to the SVC, which was seen in three out of four of our patients. Less commonly, it may connect to the brachiocephalic vein, inferior vena cava, coronary sinus, azygous vein, or right atrium. Most anomalous pulmonary veins arise mainly from the right lung. Only 3%-8% originate from the left lung [4]. A variant of PAPVR known as Scimitar syndrome in which the right-sided pulmonary vein drains into the IVC, causing hypoplasia of the right lung and right pulmonary artery. Left heart hypoplasia can also be seen in Scimitar syndrome [5].

Secundum ASD is usually present, but PAPVR can sometimes present as an isolated congenital anomaly. PAPVR associated with ASD causes a left-to-right shunt leading to an increase in flow and pressure within pulmonary vasculature causing vascular remodeling, ultimately resulting in pulmonary hypertension. Isolated PAPVR, especially those involving a single anomalous pulmonary vein, generally do not develop pulmonary hypertension unless there is a significant degree of the left-to-right shunt [6]. Most patients with isolated PAPVR remain asymptomatic.

TTE is usually the initial tool in the diagnosis of PAPVR. The diagnosis is typically confirmed by right heart catheterization (RHC) and cardiac MRI. In the modern era, computed tomography and MRI have been gaining increasing importance. CT helps in better identification of the anatomy of the pulmonary vein and its tributaries. However, patients get exposed to high radiations and data processing needs expertise and it is time-consuming [7]. MRI does not require contrast injection and has no risk of exposure to ionizing radiation. However, this modality takes longer examination times and has lesser spatial resolution compared to CT [8]. In PAPVR, RHC reveals a step up in oxygen saturation of the SVC which is greater than the brachiocephalic vein. Abnormal step up in oxygen saturation should always be followed by cardiac MRI. Besides assessing the shunt anatomy, cardiac MRI can also accurately measure the shunt fraction (Qp/Qs ratio) as well as RV size and function [9].

The management of PAPVR is individualized based on clinical presentation and degree of the left-to-right shunt. Asymptomatic patients with small left-to-right shunt due to PAPVR generally do not require surgery. Surgical repair is indicated for symptomatic patients with a hemodynamically significant left-to-right shunt (Qp/Qs >2) on cardiac MRI, such as patient 3 and patient 4, often manifesting as right ventricular volume overload. Other indications for surgery include concurrent surgical repair of other major cardiac lesions, which was the case for patient 2, or recurrent pulmonary infections [10]. Without surgical repair, PAPVR patients with Qp/Qs ratio greater than 2 may develop shunt reversal i.e. right-to-left shunt with Qp/Qs ratio less than 1. The Eisenmenger physiology usually occurs only in PAPVR patients associated with ASD. Surgery is generally contraindicated in these cases due to high surgical risks, the risk of cardiac output reduction, and the low likelihood of reversal of pulmonary vasculature remodeling [11,12]. Pulmonary artery vasodilator therapy can be considered in these PAPVR patients complicated by Eisenmenger syndrome [13].

## Conclusions

In contrast to total anomalous pulmonary venous return that is diagnosed in newborns, PAPVR patients can remain asymptomatic until the development of significant pulmonary hypertension. In PAPVR patients with or without associated ASD, the decision for surgical repair depends on symptoms, shunt fraction, recurrent pulmonary infections, and concurrent indication for cardiac surgery.
